# Dimeric structures of quinol-dependent nitric oxide reductases (qNORs) revealed by cryo–electron microscopy

**DOI:** 10.1126/sciadv.aax1803

**Published:** 2019-08-28

**Authors:** Chai C. Gopalasingam, Rachel M. Johnson, George N. Chiduza, Takehiko Tosha, Masaki Yamamoto, Yoshitsugu Shiro, Svetlana V. Antonyuk, Stephen P. Muench, S. Samar Hasnain

**Affiliations:** 1 Molecular Biophysics Group, Institute of Integrative Biology, Faculty of Health and Life Sciences, University of Liverpool, Liverpool L69 7ZB, UK.; 2 School of Biomedical Sciences, Faculty of Biological Sciences, University of Leeds, Leeds LS2 9JT, UK.; 3 Astbury Centre for Structural and Molecular Biology, University of Leeds, Leeds LS2 9JT, UK.; 4 RIKEN SPring-8 Center, 1-1-1 Kouto, Sayo, Hyogo 679-5148, Japan.; 5 Graduate School of Life Science, University of Hyogo, 3-2-1 Kouto, Kamigori, Ako, Hyogo 678-1297, Japan.

## Abstract

Quinol-dependent nitric oxide reductases (qNORs) are membrane-integrated, iron-containing enzymes of the denitrification pathway, which catalyze the reduction of nitric oxide (NO) to the major ozone destroying gas nitrous oxide (N_2_O). Cryo–electron microscopy structures of active qNOR from *Alcaligenes xylosoxidans* and an activity-enhancing mutant have been determined to be at local resolutions of 3.7 and 3.2 Å, respectively. They unexpectedly reveal a dimeric conformation (also confirmed for qNOR from *Neisseria meningitidis*) and define the active-site configuration, with a clear water channel from the cytoplasm. Structure-based mutagenesis has identified key residues involved in proton transport and substrate delivery to the active site of qNORs. The proton supply direction differs from cytochrome c–dependent NOR (cNOR), where water molecules from the cytoplasm serve as a proton source similar to those from cytochrome c oxidase.

## Teaser

qNORs that catalyze the reduction of nitric oxide to nitrous oxide are dimeric and obtain their protons from cytoplasmic end.

## INTRODUCTION

Bacterial nitric oxide reductases (NORs) are membrane-integrated, iron-containing enzymes that are involved in microbial denitrification, where soluble nitrogen oxides (e.g., nitrate) are sequentially reduced to liberate nitrogen (N_2_) into the atmosphere (*1*). NORs catalyze the reduction of two molecules of nitric oxide (NO) to nitrous oxide (N_2_O), using two protons and two electrons to cleave the N─O bond and concurrent N─N bond formation (*2*, *3*): 2NO + 2H^+^ + 2e^−^ → N_2_O + H_2_O. NORs are part of the heme-copper oxidase superfamily (HCO) (*4*) and are composed of two subdivisions based on their electron donor: cytochrome c–dependent NOR (cNOR) and quinol-dependent NOR (qNOR). cNORs have been extensively studied and consist of a complex of NorB and NorC, with the latter subunit containing heme c that acts as the electron donor to the binuclear center consisting of a high-spin heme b_3_ and a nonheme iron (Fe_B_) in NorB (*3*, *5*). Because oxygen-reducing HCO members (like cytochrome c oxidase) create a proton electrochemical gradient by pumping protons across the membrane (and by taking protons from the negative phase) (*6*, *7*), it was unexpected to find that with cNORs, the protons were taken up from the periplasmic side, indicating a non-electrogenic reaction (*8*–*10*).

In contrast, qNORs are single subunit enzymes (NorZ), in which electrons are supplied by menaquinol. qNORs are found not only in denitrifying organisms but also in various pathogenic species (*3*,*11*,*12*), many of which lack the full denitrifying apparatus, bringing to attention the role of qNORs in detoxification. NO has long been identified as a key signaling molecule, playing an important part in smooth muscle relaxation (*13*) and blood vessel dilation (*14*). NO is also produced in macrophages upon response to infectious agents (*15*), and *Neisseria meningitidis* shows depleted survival in nasopharyngeal tissue when qNOR is knocked out (*11*). NO is known to inhibit several metalloenzymes within bacteria, such as aconitase (*16*) and NADH (reduced form of nicotinamide adenine dinucleotide) dehydrogenase (*17*), and therefore, the presence of qNOR in nondenitrifying organisms seems to be that of defense rather than energy conservation. Thus, substantial efforts have been put toward structural analysis, where the x-ray crystal structure of qNOR from the thermophile *Geobacillus stearothermophilus* (*Gs*qNOR) is determined to a resolution of 2.5 Å, revealing a monomeric structure and indicating the presence of an ordered water channel toward the binuclear center from the cytoplasmic side (*18*). It has been suggested that the water channel acts in tandem with protonable residues to provide a proton-conducting channel toward the binuclear center, akin to the evolutionarily related K pathway of cytochrome c oxidases (*19*). However, the incorporation of a non-native zinc at the nonheme catalytic iron site rendered the enzyme inactive. qNOR from *N. meningitidis* (*Nm*qNOR) yielded an active enzyme, but its crystallographic structure showing a monomer could only be determined at a relatively low resolution of ~4.5 Å, where most of the side-chain configurations could not be defined (*20*).

Despite the fact that the subunit composition of cNOR and qNOR is different, there are conserved structural similarities. The NorB portion of cNOR is homologous to the C terminus of qNOR, and although it lacks heme c in the hydrophilic region, it retains a similar fold to the NorC subunit of cNOR. Both share a low-spin heme b and a high-spin heme b_3_, with a calcium ion bridging the propionates of the respective heme groups, akin to the phylogenetically related cbb_3_ oxidase (*21*). This apparent structural similarity does not extend to either the electron source or the provision of protons required for catalysis. It is hypothesized that qNORs may act in a similar manner to the bd-type quinol oxidase (*22*), where proton transfer from the cytoplasmic side is coupled to quinol oxidation (proton release) to the outside of the membrane.

We have purified highly active qNOR from *Alcaligenes xylosoxidans* (*Ax*qNOR), a Gram-negative β-proteobacterium that is equipped with denitrification enzymes, in line with its role as an environmental organism found in well water and moist soil (*23*). *A. xylosoxidans* is considered an opportunistic pathogen and has been implicated in nosocomial infections (*24*, *25*), is found in patients with cystic fibrosis (*26*), and is also thought to be a multidrug-resistant organism (*26*, *27*). Here, we have used these preparations to obtain high-resolution structures for the functional wild-type qNOR and its activity-enhanced mutant (Val^495^Ala) by single-particle cryo–electron microscopy (cryo-EM) to local resolutions of 3.7 and 3.2 Å, respectively. Cryo-EM structures unexpectedly revealed *Ax*qNOR to be a dimer. We thus determined a low-resolution (9 Å) cryo-EM structure of active *Nm*qNOR, which is also revealed as a dimer establishing the functional unit of qNORs. A structure-guided mutational study of *Ax*qNOR has allowed us to identify key residues that control and regulate proton and substrate access and thus catalysis.

## RESULTS

### Cryo-EM structural determination of *Ax*qNOR

We explored the structural determination of *Ax*qNOR by single-particle cryo-EM, given the recent achievements in solving sub–100-kDa membrane protein complexes (*28*, *29*). Although *Ax*qNOR preparations had shown a monomer-dimer equilibrium similar to other qNORs, as crystallographic structures of *Gs*qNOR and *Nm*qNOR had revealed them to be monomers [molecular weight (MW), ~85 kDa], it was considered that the *Ax*qNOR monomer on its own would be relatively small for a high-resolution EM structure determination. To facilitate the cryo-EM structural analysis of *Ax*qNOR, we prepared fusion partner constructs to increase the MW of the monomer (96 kDa) to help with the alignment of particles during processing. Guided by information from protein disorder servers, we truncated the C terminus of *Ax*qNOR and fused several different fusion partners for expression trials. *Ax*qNOR fused with apocytochrome b_562_ [BRIL (*30*)], *Ax*qNOR-BRIL, yielded membrane fractions with high NO consumption rates relative to the total protein content and was chosen for structural studies. Purified *Ax*qNOR-BRIL showed a similar NO consumption to wild-type *Ax*qNOR (284 ± 14 versus 291 ± 6 μmol NO min^−1^ μmol^−1^ qNOR). An exploratory cryo-EM dataset revealed that the particles had a preference toward the carbon edges, with only a low level of clumping present. This likely reflects the “halo” effect that some detergent-solubilized samples exhibit upon freezing, where thinner ice is observed in the center of the hole and becomes thicker toward the edge (*31*). A small test dataset of ~1200 micrographs produced an initial ~7 Å reconstruction, revealing a previously unseen dimeric form of the protein. Subsequently, a larger dataset (3213 micrographs) was collected thereafter (table S1), of which the particles produced two-dimensional (2D) class averages with visible secondary structure elements, showing a dimeric *Ax*qNOR enveloped by a large micelle. The BRIL portion was less clear, possibly due to its flexibility. Note that no monomeric qNOR classes could be generated from the large particle set, highlighting the fact that the samples are dominated by the dimeric species. Data were processed, with C2 symmetry imposed, providing a structure with a global resolution of 3.9 Å [estimated using the gold standard Fourier shell correlation (FSC) = 0.143 criterion (*32*)], where local resolution valued the core of the protein calculated at 3.7 Å. Because the BRIL density was at significantly lower resolution, it was omitted during model building, leaving just the *Ax*qNOR model.

**Fig. 1:**
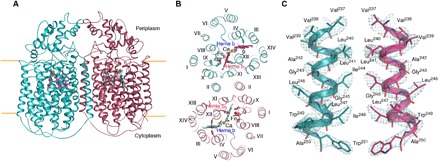
Overall structure of *Ax*qNOR and interhelical dimer interactions. (**A**) Dimeric *Ax*qNOR in plane of the lipid bilayer (orange lines) colored by chain (A in teal and B in maroon), with heme groups denoted as purple and cyan sticks. (**B**) *Ax*qNOR viewed from the periplasmic side showing transmembrane helices numbered with roman numerals, with heme and nonheme iron (Fe_B_) ligating histidines shown as sticks. Calcium is shown as green spheres, and Fe_B_ is shown as red spheres. (**C**) Hydrophobic interactions between TMHII of each respective Val^495^Ala molecule with the density map contoured at 7σ around the residues, with the wild-type exhibiting a consistent arrangement.

**Fig. 2:**
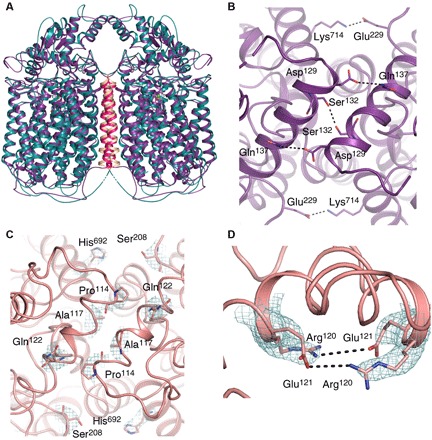
Comparison of qNOR dimers and potential soluble interface interactions. (**A**) Alignment of *Ax*qNOR (teal) and the dimeric *Gs*qNOR assembly (purple) via PISA. The structures align relatively well despite an overall RMSD of 2.35 Å; dimer mediating TMHII of *Gs*qNOR and *Ax*qNOR are colored in red and gold, respectively. (**B**) Potential interactions between the *Gs*qNOR monomers in the dimer, as determined by PISA, shown from the periplasmic side, with residues shown as purple sticks. All interactions are ≤4 Å in bond length. Most of the interactions could occur via a periplasmic helical region, with Glu^229^ and Lys^714^ located on TMHIX and TMHX of opposing molecules, respectively. (**C**) Equivalent residues in *Ax*qNOR (mutant structure) contoured with density at 5σ showing no feasible interactions. (**D**) Potential interactions between Arg^120^ and Glu^121^ of opposing *Ax*qNOR molecules, where the density was contoured at 4σ for the side chains and the Glu^121^ side chain could not be resolved.

The dimeric *Ax*qNOR is related by a twofold symmetry axis and exhibits a similar overall structure to those of other qNORs, with 14 transmembrane helices (TMHs) and an α-helical hydrophilic region (Fig. 1A). The heme b_3_ and Fe_B_ ligating ligands are present on TMHVIII, TMHIX, and TMHXII, where the heme b ligands are present on TMHXII and TMHIV (Fig. 1B). The map showed helix pitches and density for bulky side chains, consistent with the estimated resolution, with several hydrophobic interactions between TMHII of each respective molecule, where Leu^240^, Leu^241^, and Ile^244^ act to stabilize the interhelical interaction (Fig. 1C). It is evident that TMHII is a key player in maintaining the *Ax*qNOR dimer, and its importance is discussed in more detail below. In contrast, *Gs*qNOR and *Nm*qNOR were considered to be monomers in the crystal structure. Although inactive monomeric *Gs*qNOR is present in the asymmetric unit, a reexamination of the high-resolution crystallographic structure by PISA (Protein Interfaces, Surfaces and Assemblies) (*33*) shows that it also probably forms a dimer in the crystal lattice (Fig. 2A), preserving the helical interface. In addition, *Gs*qNOR shows a number of residues on the periplasmic helical region that could also contribute to the dimeric interface (Fig. 2B). The equivalent region in *Ax*qNOR revealed no such favorable interactions (Fig. 2C), despite the fact that the dimer interface area is similar to that in *Gs*qNOR (2278 Å^2^ versus 2237 Å^2^, calculated by PISA). However, several charged residues, such as Arg^120^ and Glu^121^, may interact at some point, despite the lack of Glu^121^ side-chain density (Fig. 2D). The oligomeric status of active *Nm*qNOR was assessed by obtaining a cryo-EM dataset of wild-type *Nm*qNOR. The data produced a 9 Å resolution structure, indicating the enzyme to be a dimer (fig. S1).

### Catalytic site arrangement reveals altered glutamate conformation

**Fig. 3:**
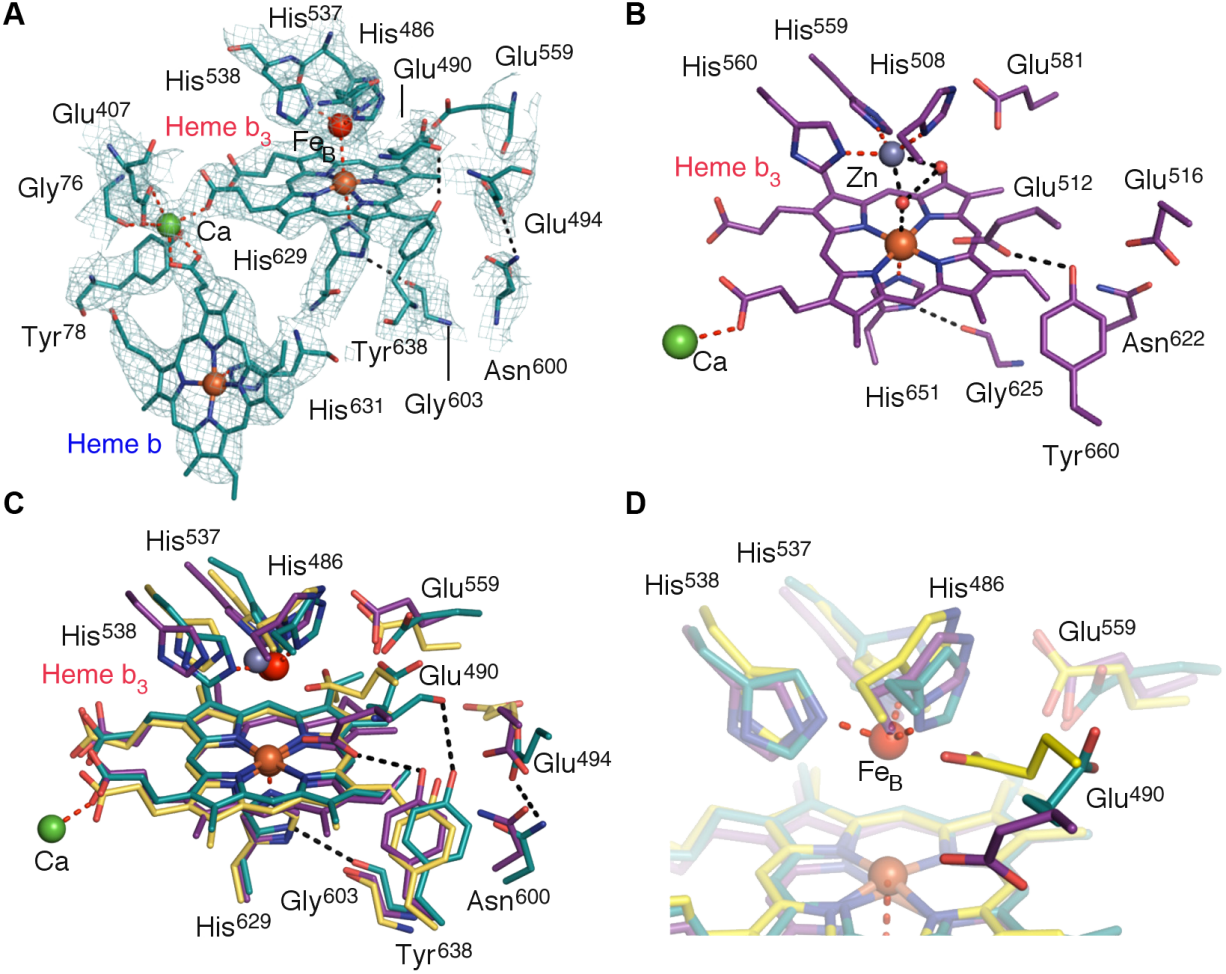
Active-site configurations in NORs. (**A**) Binuclear center and corresponding density of *Ax*qNOR with Fe_B_ (red sphere) coordinated by His^486^, His^537^, and His^538^, where Glu^490^ facing away from Fe_B_ with calcium (green sphere) is ligated by heme b and heme b_3_ propionates, Glu^407^, Try^78^, and Gly^76^ (the latter three residues are highly conserved in qNORs). Tyr^638^ and Gly^603^ provide additional structural support to the Glu^490^ backbone carbonyl oxygen and His^629^ ND1 atom, respectively. (**B**) Binuclear center of *Gs*qNOR with zinc and water molecules shown as gray and red spheres, respectively. (**C**) Comparison of side-chain conformations (numbered according to *Ax*qNOR) viewed from above the binuclear center in *Ax*qNOR (teal), *Gs*qNOR (purple), and *Pa*cNOR (yellow). Zinc is shown as a gray sphere, while Fe_B_ is colored red. (**D**) Zoomed-in view of the binuclear center, highlighting the Glu^490^ orientation of *Gs*qNOR, *Ax*qNOR, and *Pa*cNOR, colored as in (C).

The arrangement of the metal centers, namely, the electron-accepting heme b, the propionate bridging Ca^2+^, and the binuclear center of heme b_3_ and Fe_B_, is largely similar to those of *Gs*qNOR and *Nm*qNOR, aside from the heme b_3_ iron to Fe_B_ distance. The densities of heme b_3_, Fe_B_, and their respective ligands (His^486^, His^537^, His^538^, and His^629^) are well defined, where additional conserved residues (Tyr^638^ and Gly^603^) provide structural support to Glu^490^ and His^629^, respectively (Fig. 3A). Previous structures of active NORs, including cNOR, have shown the heme b_3_ Fe to Fe_B_ distance to be between 3.2 and 3.9 Å [for *Nm*qNOR (*20*) and *Pseudomonas aeruginosa* cNOR (*Pa*cNOR) (*5*), respectively], whereas in *Ax*qNOR, the distance is 4.1 Å. In *Gs*qNOR, the distance was 4.8 Å, albeit with zinc instead of iron and with the heme b having been reduced by x-ray exposure (Fig. 3B). An important area of ambiguity in previous structures of qNORs was the side-chain orientation of Glu^490^ (Glu^512^ in *Gs*qNOR) because zinc was found in the *Gs*qNOR structure (rendering it inactive) and side-chain density could not be resolved with the *Nm*qNOR structure. In *Pa*cNOR, the corresponding residue Glu^211^ ligates the nonheme iron, while in *Gs*qNOR, it is not a ligand for zinc. The *Ax*qNOR structure contains the correct nonheme metal at the binuclear center (Fe_B_), but the density for the carboxyl group of Glu^490^ suggests a different conformation for the side chain compared to *Gs*qNOR and *Pa*cNOR, with the carboxyl group approximately 3.5 Å from the His^486^ NE2 atom (Fig. 3, C and D). This may reflect a genuine difference in proton delivery pathways between the qNOR (as represented by the structure of active *Ax*qNOR) and the cNOR (as represented by the structure of active *Pa*cNOR) because the latter receives protons from the periplasm via Glu^280^ (Glu^559^ in *Ax*qNOR) (*5*).

### A 3.2 Å structure of an activity-enhanced Val^495^Ala in *Ax*qNOR

**Fig. 4:**
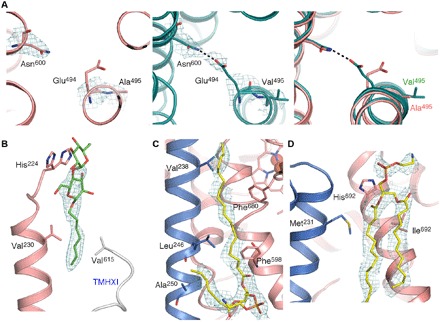
Val^495^Ala-BRIL structural features and lipid-protein interactions. (**A**) Insight into the structural impact of the Val^495^Ala mutation on the orientation of Glu^494^: left, density for Val^495^Ala contoured at 4σ with Glu^494^ side chain not visible in the density map; middle, wild-type structure density contoured at 4σ (Glu^494^-Asn^600^ salt bridge shown in black); right, aligned structures highlighting the differences between side-chain orientations (wild type in teal and Val^495^Ala in pink). (**B**) The possible truncated DDM molecule (depicted as green stick) at the dimer interface, mediated by His^224^, is shown in two conformations near the detergent head group, while highly conserved valine residues on TMHII (Val^230^ and Val^237^) and Pro^613^ from the opposing molecule (TMHXI) may stabilize the acyl chain. Symmetry-related DDM is not shown for clarity. (**C**) Phosphatidylethanolamine (PE) molecule, shown as a yellow stick, found toward the cytoplasmic end of the protein, with the acyl tail running close to TMHII residues (Val^238^, Leu^246^, and Ala^250^) of one qNOR chain (in blue) and Phe^598^ and Phe^680^ of another chain (in pink). (**D**) PE molecule (yellow stick) found on the periplasmic side of the dimer interface, stabilized by Met^231^ of TMHII, with His^692^ interacting electrostatically with the propionate group.

An analysis of residues believed to be functionally important in qNORs (vide infra) in the context of the cryo-EM structure of wild-type *Ax*qNOR was done to design and test a number of mutants. One of the mutants, Val^495^Ala, revealed an enhanced activity (by twofold) compared to wild type and was thus chosen for cryo-EM structure determination. A total of 227,000 particles were used in the final refinement (C2 symmetry), with a global resolution of 3.3 Å and a local resolution at 3.2 Å. Upon docking the wild-type EM model into the map, the mutation was clearly visible (Fig. 4A). A notable difference was the poor density of Glu^494^ side chain, which, in the wild-type *Ax*qNOR, was resolved to be facing toward Asn^600^, forming a hydrogen bond (Fig. 4A). The Val^495^Ala mutation appears to have increased conformational flexibility of Glu^494^. However, the overall structure of Val^495^Ala-BRIL is largely similar to that of the wild type, with some additional density between the dimer interface assigned as detergent molecules, which were used in solubilization [dodecyl maltoside (DDM)]. The density for the saccharide portion was clear for only half of the molecules and was left for visualization. These molecules are stabilized by Val^230^ and His^224^ of TMHII (seen in multiple conformations). Val^615^ of TMHXI from the opposing molecule also interacts with the acyl tail of the detergent, providing a way to stabilize the dimer interface (Fig. 4B). Density, which appeared to be phospholipid-like, was also seen near the dimer interface, with one phosphatidylethanolamine molecule providing an anchor between TMHII of one molecule and surrounding helices of the other molecule through Phe^598^ and Phe^680^ (Fig. 4C). In addition, a lipid molecule (truncated to fit the density) was seen closer to the periplasmic surface, where His^692^ potentially interacts between the phosphate head group and the acyl tail of the lipid, and also Met^231^ of TMHII in proximity to the acyl tail (Fig. 4D).

### Water-mediated proton transfer in *Ax*qNOR

**Fig. 5:**
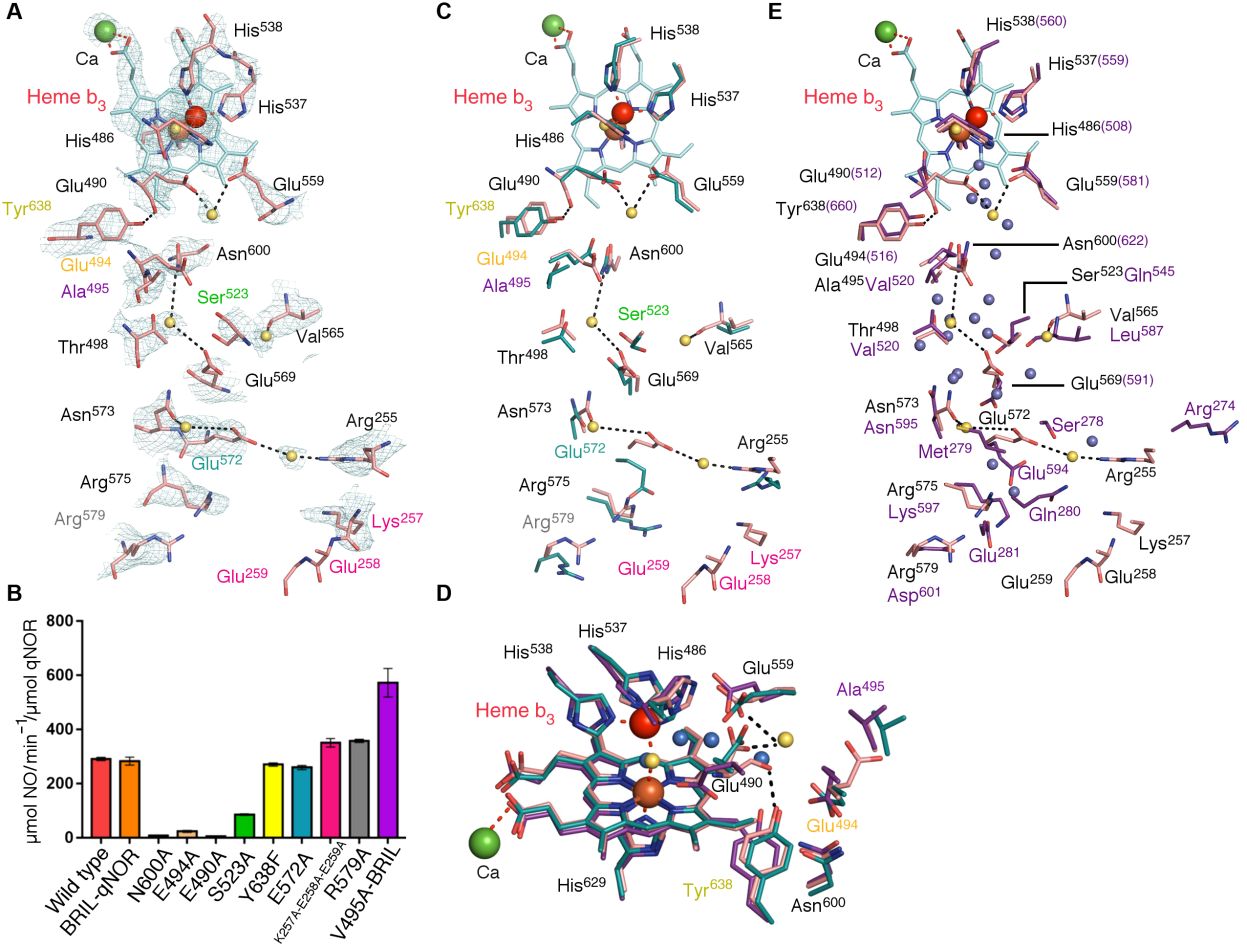
Putative water channel in *Ax*qNOR and effects of mutagenesis on NO reductase activity. (**A**) Residues [colored labels according to bar graph in (B), with the exception of Asn^600^ and Glu^490^] and accompanying densities lining the proposed proton transfer in Val^495^Ala *Ax*qNOR (pink sticks) from the cytoplasmic side to the binuclear center (calcium and Fe_B_ are shown as green and red spheres, respectively). Water molecules are shown as yellow spheres and traverse from Arg^255^ toward Glu^490^. (**B**) Bar graph detailing the effect of structure-guided mutagenesis on NO reduction rates, performed in anaerobic conditions for each variant. Colored bars indicate the mean consumption rate (*n* = 3), with SEM bars shown for each variant as black lines. (**C**) Alignment of the wild-type *Ax*qNOR (teal sticks) and Val^495^Ala proton transfer pathway indicating the variation in some side-chain conformations, notably with Glu^572^ and Glu^494^. (**D**) Comparison of the binuclear site among wild-type *Ax*qNOR (teal), Val^495^Ala *Ax*qNOR (pink), and *Gs*qNOR (purple), with residue numbers from *Ax*qNOR. Calcium is shown as a green sphere, Fe_B_ in wild-type *Ax*qNOR is shown as an orange sphere, and Fe_B_ in Val^495^Ala *Ax*qNOR Fe_B_ is shown as a red sphere. Water molecules from Val^495^Ala are depicted as yellow spheres, while those from *Gs*qNOR are shown in blue. Waters bridging the nonheme metal to the heme b_3_ (oxo ligand) and in between Glu^490^ and Glu^559^ show similar locations in both *Gs*qNOR and Val^495^Ala *Ax*qNOR structures. (**E**) *Gs*qNOR water channel (residues in purple sticks, water shown by gray spheres) aligned against Val^495^Ala *Ax*qNOR equivalent region (residues in pink, water shown as yellow spheres). Residues show higher conservation further up the channel, with *Gs*qNOR having a continuous chain of water molecules toward the binuclear site.

As a result of the improved resolution of the structure of the Val^495^Ala mutant, several new features could be resolved; most notably, a number of water molecules lining the polar channel from the cytoplasmic side to the binuclear site could be seen (Fig. 5A). Several mutants were thus generated on the basis of both sequence conservation (fig. S4) and known structural data, which were then tested for NO reduction under anaerobic conditions. Metal content analysis showed that all mutants had a minimum of 60% of Fe compared to wild type, with optical spectra of most mutants in the Soret region [maximum absorbance at 410 nm (*A*_410_)] in the oxidized state, indicative of a nonperturbed heme environment (fig. S5). *A*_410_/*A*_280_ ratios of mutants ranged from 0.73 to 0.80 (wild type at 0.70). A closer examination of a residue near the proposed proton entry site (Arg^255^) reveals a water molecule between Glu^572^ and Arg^255^, which may act as a proton entry point. Glu^572^Ala had a slightly enhanced activity compared to the wild type, which, considering its proximity to a water molecule and Arg^255^, was unexpected. Both Arg^255^ and Glu^572^ show relatively weak conservation among qNORs (fig. S6), although a charged residue usually resides in these positions. This may explain why Glu^572^Ala mutants showed little change in NO reduction activity, or it may be that Glu^572^ is bypassed during proton transfer. The sequential triple mutant in the aforementioned region (Lys^257^Ala-Glu^258^Ala-Glu^259^Ala) revealed similar consumption rates to the wild-type enzyme, ruling out any significant involvement of these residues in proton entry. Glu^258^ and Glu^259^ were not observed in the map for wild-type *Ax*qNOR, although the main chain could be resolved in the mutant map. Mutation of the highly conserved Tyr^638^ to Phe, which provides structural support to the Glu^490^ backbone carbonyl oxygen, did not severely affect the activity. In contrast, mutations of a number of residues located between the middle of the hydrophilic channel and the active site resulted in a substantial loss of activity. Ser^523^, with a conservation of ~30%, showed a ~70% loss of activity (Fig. 5B) upon mutation to alanine, which we suggest arises from the loss of a water molecule that may pass through the flexible Glu^569^ (mutants of which we could not express) and Ser^523^. Mutations of Glu^490^, Glu^494^, and Asn^600^ (based on sequence alignment of 224 qNOR sequences; the former is fully conserved, while the latter two are 99%, possibly from alignment inaccuracies due to varying gaps) resulted in over 90% loss of activity, providing evidence of their importance in qNOR functionality. The oxidized optical spectra of S523A were slightly perturbed at the Soret region, with absorbance at 411 nm (fig. S5). These are positioned along the proton delivery channel to the active site, where one of the glutamates, Glu^494^, interacts with Asn^600^ by a hydrogen bond in the wild-type structure (Fig. 5C). Glu^494^ in the 3.3 Å *Ax*qNOR-Val^495^Ala structure exhibits a loss of density, with Asn^600^ maintaining its position, yet no water molecules were found in the vicinity. The results of the Glu^494^ mutation are consistent with a previous observation that the corresponding residue in *Nm*qNOR was important for proton delivery (*20*). A water molecule was found ligating the carboxyl group of Glu^490^ and Glu^559^ (the higher-resolution *Ax*qNOR-Va^l495^Ala structure provides clear density of Glu^559^), which is not the case in *Gs*qNOR, as this water does not ligate to the equivalent residues Glu^512^ and Glu^581^ (Fig. 5D). The loss of activity for Glu^490^Ala variant in *Ax*qNOR, likely due to the loss of the water molecule and thus the proton source, is consistent with *Gs*qNOR Glu^512^Ala (*18*), although whether the Glu^512^ ligates water in the active state is not known. Overlaying the *Gs*qNOR water channel region with *Ax*qNOR-Val^495^Ala equivalent region shows a more continuous “chain” of waters in the higher-resolution (2.5 Å) *Gs*qNOR structure (Fig. 5E). Several waters are in proximity to those found in *Gs*qNOR, namely, between Glu^572^ and Arg^255^, Glu^569^ and Thr^498^, and Glu^490^ and Glu^559^, providing support to the idea that *Ax*qNOR probably uses protons from the cytoplasmic side for catalytic NO reduction.

### A putative substrate access channel

**Fig. 6:**
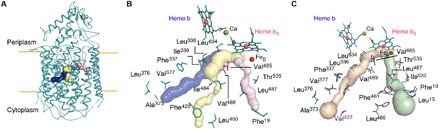
Putative NO diffusion pathways in wild-type *Ax*qNOR. (**A**) Location of proposed hydrophobic channels in wild-type *Ax*qNOR protruding from the bilayer to the binuclear site, based on CAVER analysis, shown as blue, yellow, and pink tunnels. (**B**) Location of hydrophobic channels viewed from the periplasmic side lined with residues situated along the tunnels (heme groups are shown as teal sticks, with calcium and Fe_B_ shown as green and red spheres, respectively). Val^485^ and Val^489^ are colored in pink and purple sticks. (**C**) *Gs*qNOR hydrophobic tunnels [from Matsumoto *et al.* (*18*)] with residue numbers from *Ax*qNOR (teal) and *Gs*qNOR (purple) aligned, showing similar spatial location and residue type, with Val^485^ being replaced with Ile^507^ in *Gs*qNOR.

We conducted a search of potential channels within our *Ax*qNOR structures using CAVER (*34*) to reveal whether any relevant channels could pertain to substrate entry and delivery to the active site. Starting the search from the active site, three potential candidates were found to be plausible (Fig. 6, A and B), two of which pass by Val^485^ (~7 Å from Fe_B_) en route to the active site from the bilayer. Given the knowledge that Val^206^ in *Pa*cNOR is implicated in NO transport and is found in the same spatial position as Val^485^, we mutated Val^485^ to alanine to ascertain whether it can facilitate NO transport or inhibit it. The Val^485^Ala mutant of *Ax*qNOR exhibited a ~70% loss of activity, signifying its role in NO delivery. The substitution to a smaller side chain may be expected to widen the channel, potentially allowing less constraint for NO to access the catalytic site. Yet, the Val^206^Trp mutant of *Pa*cNOR caused loss of NO consumption in vitro, which is suggested to be due to the increase in bulk, creating a barrier near the active site (*35*). Having a residue of sufficient hydrophobicity and appropriate size is a key component in determining NO access to the active site. Closer inspection of the structure showed that the highly conserved Val^489^ (fig. S4) lies beneath Val^485^, which may act in tandem with Val^485^ to funnel NO toward the active site. *Gs*qNOR channel analysis revealed two channels that overlap a similar spatial location in *Ax*qNOR, which may indicate a conserved role of these hydrophobic tunnels in substrate transfer (Fig. 6C). Comparison with structures of *Pa*cNOR [and its complex with *cd_1_*NiR (*35*)] and *Thermus thermophilus* cytochrome ba_3_ (*36*) suggest that similar hydrophobic channels exist for gas diffusion from outside the membrane to the active site.

## DISCUSSION

Highly active preparations of *Ax*qNOR were obtained but did not yield well-diffracting crystals <7 Å, despite substantial efforts. Given a relatively small MW of the monomer (~85 kDa) for cryo-EM experiments, it was fused with apocytochrome b_562_ [BRIL(*30*)], raising the MW of the monomer to ~96 kDa. This facilitated structural determination by cryo-EM, unexpectedly revealing a dimeric *Ax*qNOR (predicted MW with BRIL, ~190 kDa). The high quality of the structures suggests that BRIL may have also stabilized *Ax*qNOR, something often seen in the crystallographic structure determination of G protein (heterotrimeric guanine nucleotide–binding protein)–coupled receptors (*37*). Attachment of BRIL at the C terminus of *Ax*qNOR (TMHXIV) did not alter the enzymatic activity. Despite the high quality of maps, BRIL could not be resolved to high resolution, although it is clear from both the 2D classes and 3D reconstruction that its spatial location is well away from the dimer interface and it displays a large degree of flexibility (fig. S2). The resolution obtained here compares favorably against complexes of larger sizes, using a similar number of particles in the final refinement (52,000 to 57,000), e.g., cytochrome bc_1_ (~500 kDa, 4.4 Å) (*38*) and alternative complex III (~316 kDa, 3.9 Å) (*39*). The Val^495^Ala-BRIL mutant provided a structure at a higher resolution of 3.3 Å using 227,000 particles, with local resolution estimates from ResMap (*40*) ranging to as high as 2.5 Å (fig. S3).

The dimeric structure of a qNOR observed here raised an important question regarding the oligomeric status of the functionally important form of the previously crystallographically studied qNORs. A cryo-EM data collection of wild-type *Nm*qNOR provided a 9 Å structure demonstrating it to be dimer, suggesting that crystallization may have preferentially selected the monomeric species. Both *Ax*qNOR cryo-EM structures show that TMHII, which is absent in cNORs, maintains the dimeric form of *Ax*qNOR with several hydrophobic interactions between TMHII of each respective molecule, acting to stabilize the interhelical interaction. Lipids are known to facilitate and be indispensable for oligomerization and functioning of several membrane protein complexes [e.g., leucine transporter (*41*), cytochrome c oxidase (*42*), and cytochrome bc_1_ (*43*)]. The presence of lipids around the dimer interface may be a key physiological feature in qNORs, and the presence of detergent at the dimer boundary might be a surrogate for host lipids, because the hydrophobic residues on TMHII and Val^615^ from the opposing molecule interacted with the acyl tail and could conceivably do so with lipids. Because monomeric qNOR is also isolated during purification, the addition of detergent may replace some weakly bound lipids, which may alter the dimer affinity, causing a greater propensity of monomers or preferential selection during crystallization conditions. The absence of monomers in the cryo-EM data suggests that dominant species of active *Ax*qNOR and *Nm*qNOR, from which EM grids were made, is dimeric.

Although the replacement of nonheme Fe_B_ with zinc in *Gs*qNOR hampered the elucidation of a structure-function relationship in qNOR, comparing the structure of inactive *Gs*qNOR with that of active *Ax*qNOR provides valuable information in reaching the structure-property relationship that is generic for qNORs. A proton entry site for qNORs has been proposed to be on a cytoplasmic loop, composed of several charged residues. Our triple mutant (Lys^257^-Glu^258^A-Glu^259^A) had a similar activity to the wild type, which was similar in the case of *Nm*qNOR with the Glu^259^ point mutations. The enhanced resolution for *Ax*qNOR from the structure of the Val^495^Ala-BRIL mutant has allowed us to map out a number of water molecules, with Arg^255^ potentially acting as a starting point for proton transfer from the cytoplasmic solvent toward the binuclear site. The significantly enhanced activity of Val^495^Ala is likely to arise from alleviating proton transfer as the rate-limiting step. This most likely results from increased conformational dynamics of Glu^494^, which shuttles the proton toward the catalytic pocket. The importance of several residues located between the binuclear center and the cytoplasmic surface is indicated in this study, namely, Ser^523^ and Asn^600^, in maintaining a putative proton transfer channel with possible partners Glu^569^ and Glu^494^, respectively. Ser^523^, despite its relatively low conservation (30% among 224 sequences), exhibits a significant loss of activity upon mutation. The conformer of Glu^569^ is facing toward Ser^523^ in our structures, which may indicate a functional role of Glu^569^-Ser^523^ in proton transport. The drastic effect on activity due to the mutation of Glu^494^ in both *Ax*qNOR and *Nm*qNOR (Glu^498^) suggests its critical functional role in qNOR. However, the equivalent mutant in *Paracoccus denitrificans* cNOR (Glu^202^) shows only ~60% loss of activity when heterologously expressed *in Escherichia coli* JM109 cells (*44*, *45*), consistent with its potential role in providing a sufficient electronegative environment to lower the redox potential of the heme b_3_ iron compared to the other heme groups (*46*). It should be noted that Glu^490^ and Glu^494^ variants in *Ax*qNOR exhibited ~70% of Fe compared to wild type. This may contribute to a lower activity, but the oxidized and reduced optical spectra of all variants were identical to those of wild type (fig. S5), suggesting that iron is not depleted from the Fe_B_ site preferentially. Contrary to cNOR, proton transfer pathway in qNORs likely originates from the cytoplasmic side, with Glu^494^ playing an important part in proton transport.

The mechanism of NO reduction in membrane-integrated NORs has been the subject of intense investigation, and the structural determination of *Pa*cNOR was a significant step. A key element was the proposed flexibility of Glu^211^ (coordinating Fe_B_) to accommodate two NO molecules in the active site. Glu^211^ (Glu^490^ in *Ax*qNOR) is proposed to accept protons from Glu^280^ (Glu^559^ in *Ax*qNOR), causing protonation and possible dissociation from Fe_B_, allowing NO binding [assuming that an increase in the Fe-Fe distance to at least 4.4 Å occurs, as suggested by Richardson and colleagues (*2*)], while structures of the ligand analog–bound forms of *Pa*cNOR showed that Glu^211^ coordinated to Fe_B_ even in the presence of the ligand. Glu^490^ in *Ax*qNOR unequivocally faces away from Fe_B_, suggesting that Glu^490^ and its equivalents in (active) qNORs do not act as an Fe_B_ ligand and instead ligate a water molecule along with Glu^559^. The directionality of proton transfer to the terminal glutamates in qNOR differs from cNOR, as Glu^494^ in *Ax*qNOR is crucial to activity but the equivalent glutamate in cNOR only loses half its activity when mutated (*44*), possibly down to periplasmic supplied protons flowing through Glu^280^ toward Glu^211^. The precise role of this residue in cNOR is still unclear. It has been suggested that it may be a remnant in the evolutionary pathway in acquiring protons from the cytoplasmic end in cytochrome oxidases because it is (roughly) positioned at the end of the K pathway (*47*).

Molecular dynamic simulations of the crystal structure of *Pa*cNOR-*cd_1_* NiR complex suggested that NO transfer may occur primarily via the lipid bilayer and not via the active sites of each enzyme (*35*). Given the lipophilicity of NO, the migration of NO toward and into the lipid bilayer is feasible. Depending on where *Ax*NiR may interact (with either *Ax*qNOR, the lipid bilayer, or both), the use of either channel seems plausible for providing access to NO released from *Ax*NiR. We identified Val^485^ to be an important residue in guiding NO to the active site of *Ax*qNOR, but as the Val^485^Ala mutant exhibited a loss of 70% activity, additional inputs may be present. In view of the need to detoxify NO rapidly, having multiple entry points may be an advantage. The passage of NO and its binding to qNOR may also need to be barrier-less, consistent with a fast rate constant as established in cNOR (*48*). The tunnel analysis suggests that Leu^376^ and Phe^420^ may act as input points, yet the intersections of the tunnels before the active site consist of Val^485^ and Val^489^. Because Val^489^ is strongly conserved (fig. S6), it may compensate for the loss of Val^485^ to continue shuttling NO. A computation study on *Pa*cNOR investigated NO diffusion and found that several migration pathways exist (termed dominant and alternate), where migration of NO is ~10-fold lower using the alternate route than using the dominant pathway (*49*). Several of the routes include conserved residues found in our tunnel analysis (Val^285^, Ile^484^, Leu^336^, and Leu^487^), which may suggest a generic role in NO transport across NORs. A similar study on cytochrome ba_3_ found hydrophobic tunnels that are located within the bilayer and provide O_2_ an area to partition from the aqueous phase, with no significant energetic barriers for O_2_ transport (*50*).

In summary, we solved the structure of wild-type and mutant *Ax*qNOR at local resolutions of 3.7 and 3.2 Å, respectively, by cryo-EM. These structures are the first example that documents dimeric structures of qNORs, providing details on the structural architecture. The active site and putative proton transfer pathway arrangements are revealed at a high enough resolution of an active qNOR. The current structure and mutational results, together with the previous data, suggest that qNORs take up protons from cytoplasmic side, with several water molecules facilitating this to occur. This is in contrast to cNORs, which receive protons from the periplasmic side (*5*). This redefines the role of qNOR as a respiratory enzyme, and its evolutionary relationship to cytochrome c oxidase, setting qNORs apart from both cNOR and the denitrification enzymes NiR and nitrous oxide reductase, which are not considered to be directly involved in energy conservation.

## MATERIALS AND METHODS

### Construction of *Ax*qNOR-BRIL expression plasmid and site-directed mutants

The *Ax*qNOR-BRIL and Val^495^Ala-BRIL expression plasmid was made by GenScript (Hong Kong), with the *Ax*qNOR gene (NorZ) truncated (Δ747 to Δ763) to accommodate the apocytochrome b_562_ [BRIL_562_, Protein Data Bank (PDB) accession code: 1M6T] fusion partner at the C terminus of *Ax*qNOR. The chimeric gene (qNOR-BRIL) was then ligated between the Nde I and Xho I sites of a pET-26b (+) plasmid, allowing a hexa-histidine tag to be attached at the C terminus of BRIL_562_. Site-directed mutants of qNOR were generated using the QuikChange II Kit (Agilent) using the wild-type *Ax*qNOR plasmid as a template vector. Mutations were confirmed by DNA sequencing before use.

### Purification of *Ax*qNOR-BRIL, Val^495^Ala-BRIL, and site-directed mutants

C41 (DE3) *E. coli* cells (Lucigen) were transformed with the expression plasmid and grown in 2xYT media. Upon reaching an *A*_600_ of ~2, ∂-aminolevulinic acid (Sigma-Aldrich) and FeCl_3_ were added at final concentrations of 200 μM, as well as IPTG (isopropyl-β-D-thiogalactopyranoside) was added at a final concentration of 500 μM, to induce overexpression of qNOR-BRIL. Harvested cells were washed in 50 mM tris (pH 7.0) and 150 mM NaCl and then lysed by sonication, before collecting membrane fractions by ultracentrifugation. Membranes (final concentration of 7 mg/ml) were solubilized in 50 mM tris (pH 7.0), 150 mM NaCl, and 1% (v/v) β-DDM (Anatrace) for 2 hours at 4°C. The solubilized material was separated by centrifugation at 40,000 rpm and loaded onto a pre-equilibrated 5-ml HisTrap column (GE Healthcare). The column was subjected to three column volumes worth of washing in 50 mM tris (pH 7.0), 150 mM NaCl, 20 mM imidazole, 0.05% DDM, and then two-column volumes in the same buffer, yet with 35 mM imidazole. The protein was eluted in the same buffer, using 150 mM imidazole instead. Fractions were assessed for purity by SDS–polyacrylamide gel electrophoresis and ultraviolet (UV)–visible spectroscopy (U-3300, Hitachi), with elutions having an *A*_410_/*A*_280_ ratio of >0.6 being carried forward. After concentration in Amicon Ultra 100K concentrators, qNOR-BRIL was loaded onto a Superdex 200 10/300 Increase (GE Healthcare) column equilibrated in 50 mM tris (pH 7.0), 150 mM NaCl, and 0.05% (v/v) decyl-thio-maltoside (DTM) (Anatrace). Fractions with *A*_410_/*A*_280_ > 0.7 were concentrated to 20 mg/ml and flash-frozen in liquid nitrogen, before storage at −80°C. Val^495^Ala-BRIL was purified in the same manner, except that buffers were at pH 7.5. Site-directed mutants were purified in a similar manner to *Ax*qNOR-BRIL, except that, at gel filtration, 0.05% DDM was used instead of DTM.

### UV-visible absorption spectroscopy

Absorption spectra were recorded on U-3300 spectrophotometer (Hitachi). *Ax*qNOR samples were diluted in gel filtration buffer and subjected to measurements for the oxidized state. An excess of sodium dithionite was added to the oxidized samples (final concentration, ~1 mM) and remeasured to obtain fully reduced spectra.

### Enzymatic activity measurements

Before enzyme activity measurements, samples were subjected to inductively coupled plasma optical emission spectrometry (ICP-OES) analysis with an ICP-OES 5110 instrument (Agilent) to ascertain metal content. qNOR activity was measured using a Clark-type electrode fitted with an ISO-NO Mark II system (WPI). All reaction components (aside from glucose oxidase and catalase) were made anaerobic by replacing the atmosphere of the vials with N_2_. The assay buffer contained 50 mM Na-citrate (pH 6.0), 0.05% DDM or DTM, 100 mM d-glucose, 10 μg ml^−1^ glucose oxidase, and 10 μg ml^−1^ catalase. Glucose, glucose oxidase, and catalase were added to scavenge any oxygen left in the reaction vessel. Sodium ascorbate (1 mM) and phenazine methosulfate (10 μM) acted as an electron donation system. A 2 mM NO saturated solution [50 mM Na-citrate (pH 6.0)] was made and added at a final concentration of 20 μM. NO consumption was started by addition of the protein at a final concentration of 0.2 μM.

### Cryo-EM sample preparation and data collection

qNOR-BRIL was diluted to 3 mg/ml, and 3 μl of aliquots was applied on glow-discharged Quantifoil Au R1.2/1.3 holey carbon grids. Grids were plunge-frozen in liquid ethane using Vitrobot Mark IV (FEI), with grids blotted for 6 s, and a blot force of 6, maintained at 100% humidity and 4°C. Grids were then loaded into an FEI Titan Krios TEM (Astbury Biostructure Laboratory, University of Leeds), operating at 300 kV, equipped with a K2 Summit detector (Gatan). Automated data collection was performed with EPU software at a magnification of ×75,000, using a defocus range of −1.5 to −3.5 μm. A total of 3213 micrographs were collected with a pixel size of 1.07 Å. A total dose of 65 e^−^/Å^2^ was acquired by using a dose rate of 6.21 e^−^ pixel^−1^ s^−1^ across 40 frames for 12 s of total exposure time. *Ax*qNOR Val^495^Ala-BRIL was prepared in the same way as the wild type, except that the sample (5 mg/ml) was used with Quantifoil Cu R1.2/1.3 holey carbon grids. Val^495^Ala-BRIL grids were loaded into an FEI Titan Krios at Electron Bio-Imaging Centre (eBIC) (Diamond Light Source) operating at 300 kV, equipped with a K2 Summit detector (Gatan). Automated data collection was performed with EPU software at a magnification of ×47,710, using a defocus range of −1 to −3 μm. A total of 1803 micrographs were collected with a pixel size of 1.05 Å. A total dose of 49 e^−^/Å^2^ was acquired by using a dose rate of 4.16 e^−^ pixel^−1^ s^−1^ across 40 frames for 10 s of total exposure time.

### Image processing

All image processing was performed in RELION 3.0(*51*). Beam-induced motion and drift correction were performed using RELION 3.0 own implementation. Contrast Transfer Function (CTF) estimation was carried out using CTFFIND-4.1 (*52*), with CTF correction carried out on non–dose-weighted micrographs, with further data processing done on dose-weighted micrographs. Around 2000 particles were manually picked and extracted to generate initial 2D classes that were used as templates for automatic particle picking. Approximately 700,000 particles were picked and sorted before 2D classification on ~540,000 particles was carried out. The best 2D classes were chosen to make an initial model, which was then low pass–filtered to 60 Å and used for 3D classification. Several rounds of 3D classification were performed, with the best class out of three being taken forward for the next round of classification. A total of 56,134 particles yielded a refined map at a global resolution of 4.1 Å with C2 symmetry, using a soft mask encompassing the protein and detergent micelle. CTF refinement and Bayesian particle polishing improved the resolution to 3.9 Å, where local resolutions suggest that the core of the protein is 3.7 Å. Tighter masks failed to increase the resolution of the final map. Resolutions were estimated by the gold standard FSC = 0.143 criterion, while local resolutions were valued using RELION. Val^495^Ala was processed in a similar fashion to the wild type, albeit with motion correction carried out using RELION 3.0 implementation. From ~600,000 auto-picked particles, multiple rounds of 2D and 3D classification led to 227,000 particles being used in the final reconstruction to attain a resolution of 3.7 Å, with C2 symmetry. After CTF refinement and Bayesian particle polishing, the resolution improved to 3.5 Å. A tight mask excluding the micelle was used in postprocessing to improve the resolution further to 3.3 Å, with a local resolution at 3.2 Å. Resolutions were estimated by the gold standard FSC = 0.143 criterion, while local resolutions were valued using RELION. ResMap (*40*) was used to assess slice through volume local resolution estimates.

### Model building, refinement, and validation

The high-resolution *Gs*qNOR (PDB accession code: 3AYF) structure (33% sequence identity) was fitted into the locally filtered map using the Chimeras “Fit in Map” function (*53*), which then served as a template to manually build the *Ax*qNOR structure in *Coot*. The Arp/Warp web server (https://arpwarp.embl-hamburg.de/) was used as an additional aid in manual model building in difficult regions (solvent-exposed loops and periplasmic helical region). As the density of the BRIL was far less detailed than that of the qNOR molecules, only qNOR was built into the maps. The resulting structure was then refined with secondary structure and custom geometry restraints around the metal centers (heme b, Ca^2+^, heme b_3_, and Fe_B_) and their respective ligands, using the phenix.real_space_refine program in the Phenix suite (*54*). Models were checked in *Coot* to identify any regions that needed correcting, based on both *Coot* and MolProbity validation statistics. For Val^495^Ala, B-factor sharpening was performed on the local resolution filtered map, using a value of −147 Å^2^, determined from running the bfactor_plot.py script in RELION 3.0. The structure of Val^495^Ala was solved by fitting the wild-type AxqNOR model into the map and adjusting/refining the model as described before. Figures were made using Chimera and PyMOL.

## Supplementary Material

http://advances.sciencemag.org/cgi/content/full/5/8/eaax1803/DC1

Download PDF

Dimeric structures of quinol-dependent nitric oxide reductases (qNORs) revealed by cryo–electron microscopy

## SUPPLEMENTARY MATERIALS

Supplementary material for this article is available at http://advances.sciencemag.org/cgi/content/full/5/8/eaax1803/DC1 Fig. S1. 3D cryo-EM reconstruction of wild-type *Nm*qNOR. Fig. S2. Summary of cryo-EM data collection for wild-type and Val^495^Ala *Ax*qNOR. Fig. S3. Henderson-Rosenthal plots of qNOR datasets and local resolution slice through plots. Fig. S4. Multiple sequence alignment of selected qNORs. Fig. S5. Oxidized and dithionite reduced spectra of selected *Ax*qNOR variants. Fig. S6. Residue probability chart of qNORs. Table S1. Cryo-EM data collection parameters and refinement statistics. Table S2. *Ax*qNOR putative proton transfer channel site-directed mutants’ conservation and relative activities.
