# Supplementary material for: Dimeric structures of quinol-dependent nitric oxide reductases (qNORs) revealed by cryo–electron microscopy
Source: Sci Adv. 2019 Aug 28;5(8):eaax1803. doi: 10.1126/sciadv.aax1803 (PMC6713497; doi:10.1126/sciadv.aax1803)
Supplement: http://advances.sciencemag.org/cgi/content/full/5/8/eaax1803/DC1 [file supp_5_8_eaax1803__index.html]

Science Advances | Science AdvancesAAASSearchScience AdvancesMenu

## Supplementary Materials

**This PDF file includes:**

- Fig. S1. 3D cryo-EM reconstruction of wild-type *Nm*qNOR.
- Fig. S2. Summary of cryo-EM data collection for wild-type and Val495Ala *Ax*qNOR.
- Fig. S3. Henderson-Rosenthal plots of qNOR datasets and local resolution slice through plots.
- Fig. S4. Multiple sequence alignment of selected qNORs.
- Fig. S5. Oxidized and dithionite reduced spectra of selected *Ax*qNOR variants.
- Fig. S6. Residue probability chart of qNORs.
- Table S1. Cryo-EM data collection parameters and refinement statistics.
- Table S2. *Ax*qNOR putative proton transfer channel site-directed mutants’ conservation and relative activities.

Download PDF

**Files in this Data Supplement:**

- Adobe PDF - aax1803\_SM.pdf
