# Supplementary material for: Dimeric structures of quinol-dependent nitric oxide reductases (qNORs) revealed by cryo–electron microscopy
Source: Sci Adv. 2019 Aug 28;5(8):eaax1803. doi: 10.1126/sciadv.aax1803 (PMC6713497; doi:10.1126/sciadv.aax1803)
Supplement: Download PDF [file aax1803_SM.pdf]

Science Advances
AAAS

advances.sciencemag.org/cgi/content/full/5/8/eaax1803/DC1

Supplementary Materials for

Dimeric structures of quinol-dependent nitric oxide reductases (qNORs) revealed by cryo–electron microscopy

Chai C. Gopalasingam, Rachel M. Johnson, George N. Chiduza, Takehiko Tosha, Masaki Yamamoto, Yoshitsugu Shiro, Svetlana V. Antonyuk, Stephen P. Muench, S. Samar Hasnain*

*Corresponding author. Email: s.s.hasnain@liv.ac.uk

Published 28 August 2019, *Sci. Adv.* **5**, eaax1803 (2019)
DOI: 10.1126/sciadv.aax1803

This PDF file includes:

Fig. S1. 3D cryo-EM reconstruction of wild-type *Nm*qNOR.
Fig. S2. Summary of cryo-EM data collection for wild-type and Val^{495}Ala *Ax*qNOR.
Fig. S3. Henderson-Rosenthal plots of qNOR datasets and local resolution slice through plots.
Fig. S4. Multiple sequence alignment of selected qNORs.
Fig. S5. Oxidized and dithionite reduced spectra of selected *Ax*qNOR variants.
Fig. S6. Residue probability chart of qNORs.
Table S1. Cryo-EM data collection parameters and refinement statistics.
Table S2. *Ax*qNOR putative proton transfer channel site-directed mutants' conservation and relative activities.

Figure S1

A

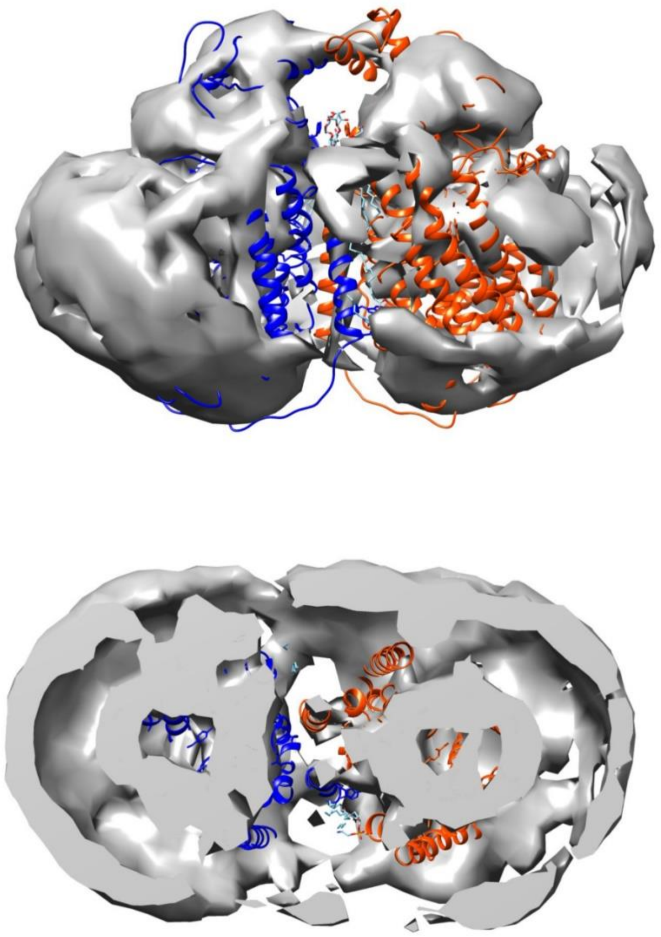

Fig. S1. 3D cryo-EM reconstruction of wild-type *Nm*qNOR. (**A**) 9 Å map generated from ~ 60,000 particles, clearly showing a dimeric form of the enzyme, having docked the *Ax*qNOR EM structure into the map (top view from the membrane plane, bottom view from the periplasmic side). Though the map has density inside of the micelle, the resolution isn't good enough to see the separation of the helices, although TMHII density aligns well with *Ax*qNOR TMHII.

Figure S2

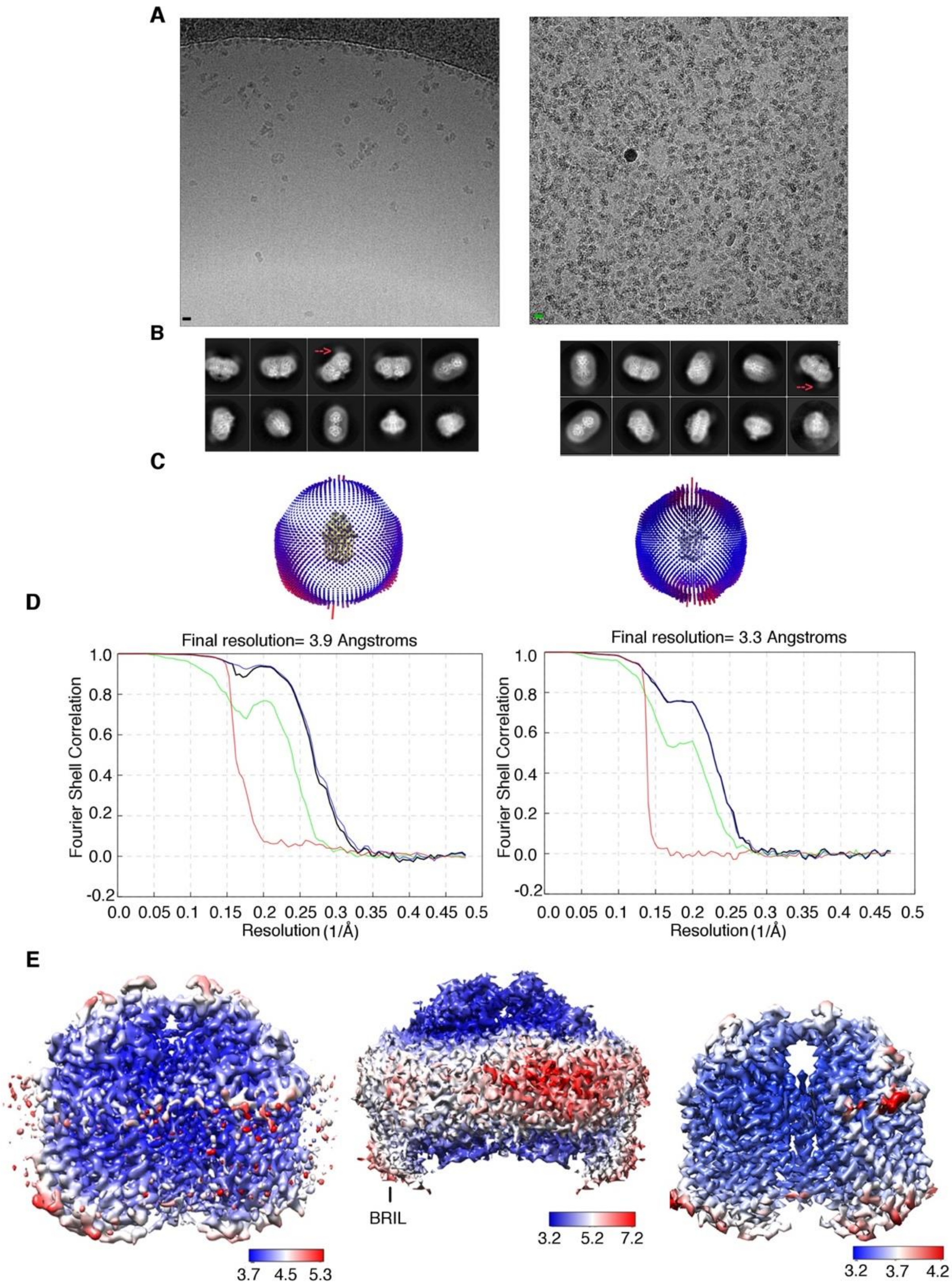

Fig. S2. Summary of cryo-EM data collection for wild-type and Val^{495}Ala *Ax*qNOR. (**A**) A representative micrograph from wildtype *Ax*qNOR data collection (left), showing particles distributed closer to the carbon edge (Scale bar= 20 nm) and from Val495Ala (right) which exhibited higher particle density due to thicker vitreous ice upon freezing (green scale bar= 20 nm). (**B**) 2D classes of wildtype (left) and Val495Ala (right) generated in RELION showing varied orientations of the *Ax*qNOR-BRIL particles, showing secondary structure elements

and a prominent detergent/lipid belt. BRIL can be seen as a fuzzy cloud on the ends of the particles (indicated with a red dashed arrow). (**C**) Angular distribution of particles used to generate the 3D wildtype map (left) and Vala495Ala map (right) after refinement with C2 symmetry. (**D**) FSC curves of wildtype (left) and Val495Ala (right), with curves colored black, green, blue and red for the corrected map, unmasked map, masked map and phase randomized map, respectively. (**E**) Left, refined 3D reconstruction of wildtype *Ax*qNOR (3.9 Å) with local resolution estimates shown as a color key, middle, refined 3D reconstruction of Val495Ala at lower threshold to show the BRIL molecules at significantly lower resolution (indicated by color key). BRIL is located on TMH XIV, away from the dimer interface, right, Val495Ala refined 3D reconstruction colored by local resolution.

Figure S3

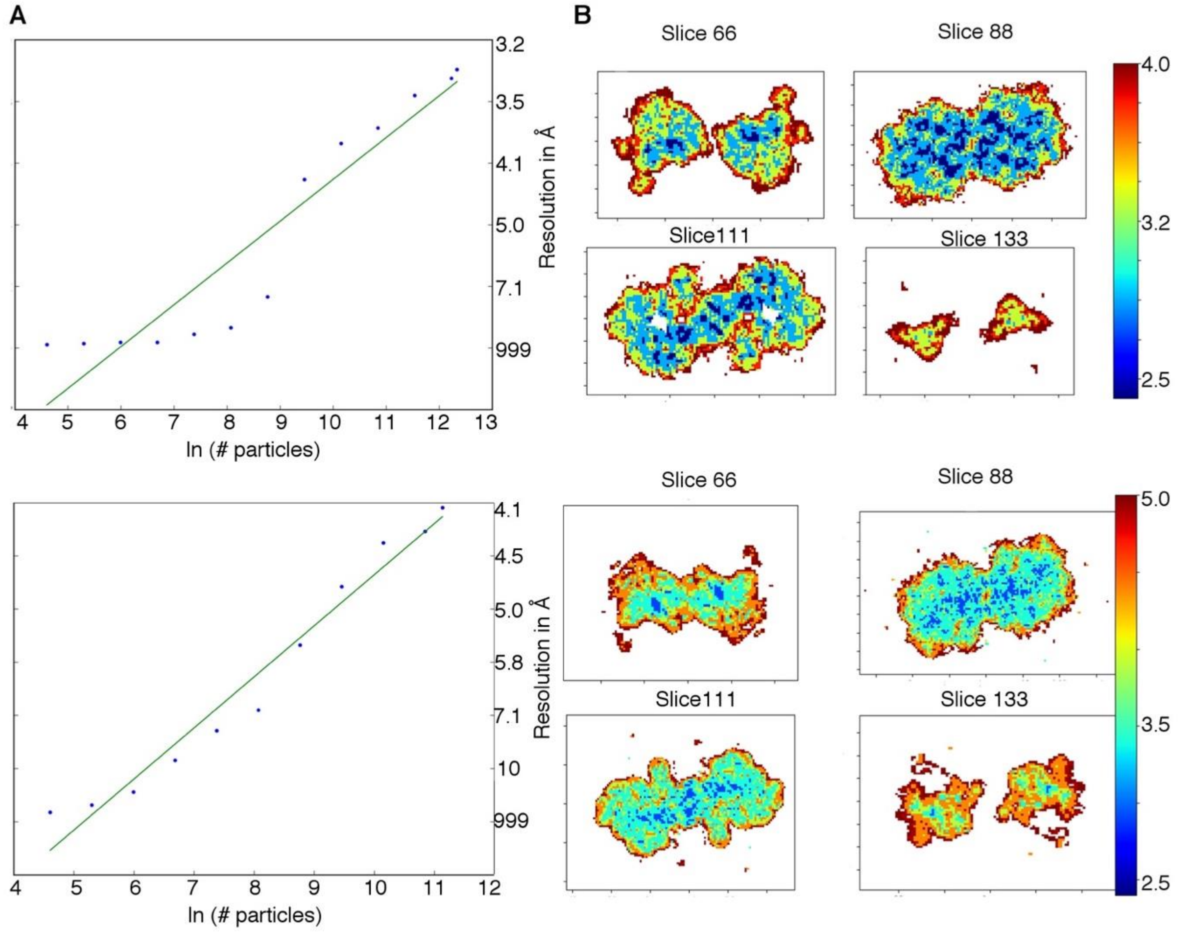

Fig. S3. Henderson-Rosenthal plots of qNOR datasets and local resolution slice through plots. (**A**) Henderson- Rosenthal plots of Val495Ala (top) and wildtype (bottom) run by RELION 3.0 *bfactor_plot* script against the refined data, using the tight mask originally used to obtain a 3.3 Å postprocessed map for Val495Ala. Blue dots represent subsets of particles used in each refinement, increasing logarithmically until the full particle set is used. (**B**) RESMAP[38] generated local resolution slice throughs of the 3D reconstruction, travelling from the periplasmic side (slice 66) to the cytoplasmic side (slice 133) of Val495Ala (top) and wildtype (bottom), with searches for maximum and minimum resolution at two and four times the pixel size of each dataset. Resolution is colored according to the key on the right. Wildtype plots show some micelle contribution due to a softer solvent mask used during postprocessing.

Figure S4

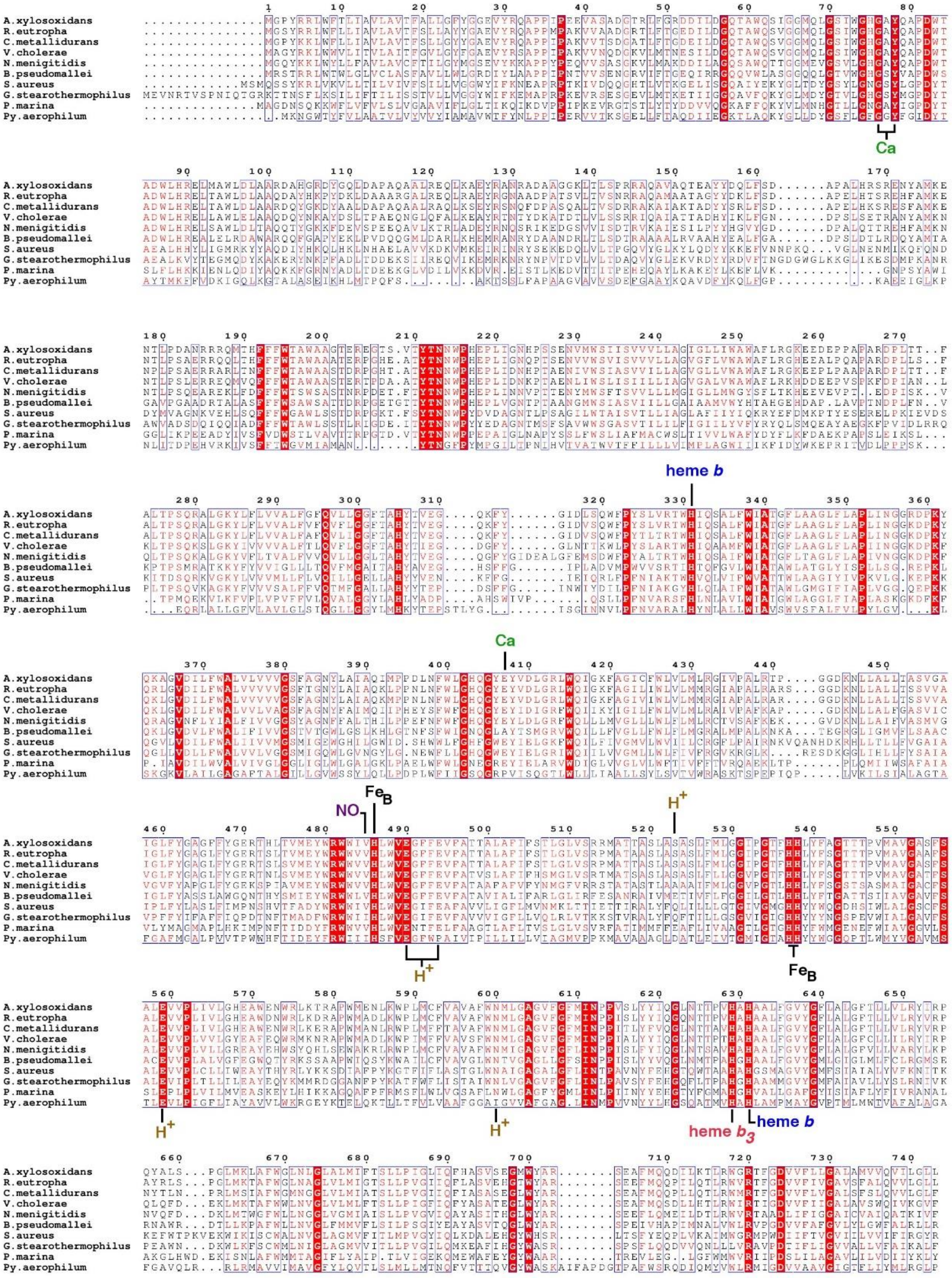

Fig. S4. Multiple sequence alignment of selected qNORs. qNOR's from various pathogenic and denitrifying species sequences were found on UniProt, with the alignment produced by Clustal W and ESPript 3.0. Sequences were truncated to residue 743 (number by *Ax*qNOR) since we could only build the *Ax*qNOR structures up till residue 743. Annotations indicate residues involved in metal binding, putative proton and NO transfer as tested in our study.

Figure S5

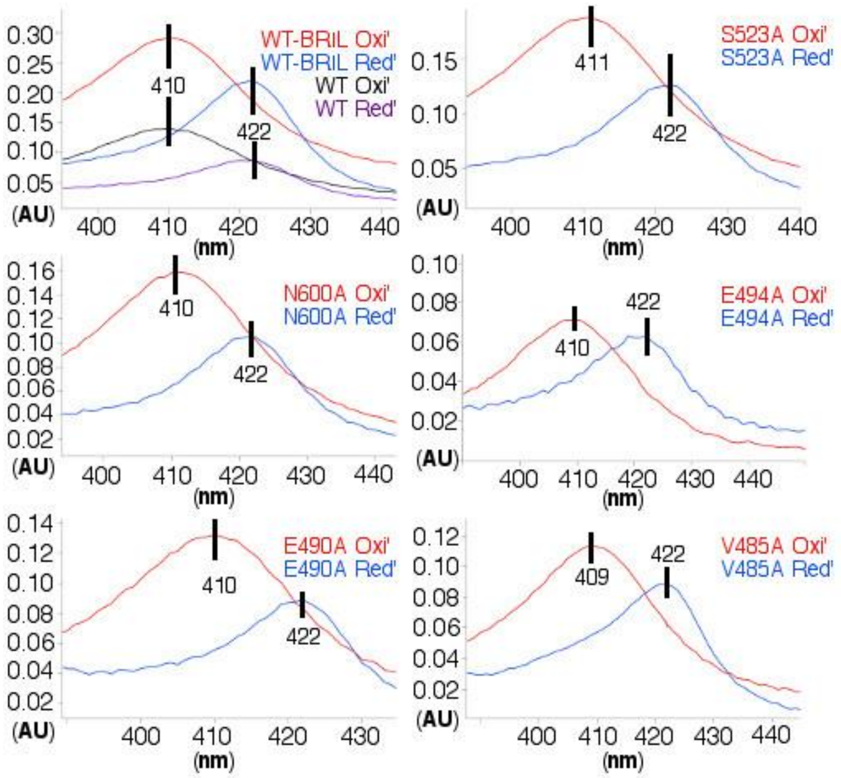

Fig. S5. Oxidized and dithionite reduced spectra of selected *Ax*qNOR variants. Optical spectra of as isolated (Oxi') and sodium dithionite reduced (Red') *Ax*qNOR variants and wildtype samples. Samples were reduced using 1 mM sodium dithionite. *Ax*qNOR samples were diluted prior to use in 50 mM Tris pH 7.0, 150 mM NaCl, 0.05 % (v/v) DDM for all variants apart from WT and WT-BRIL (0.05 % (v/v) DTM in place of DDM).

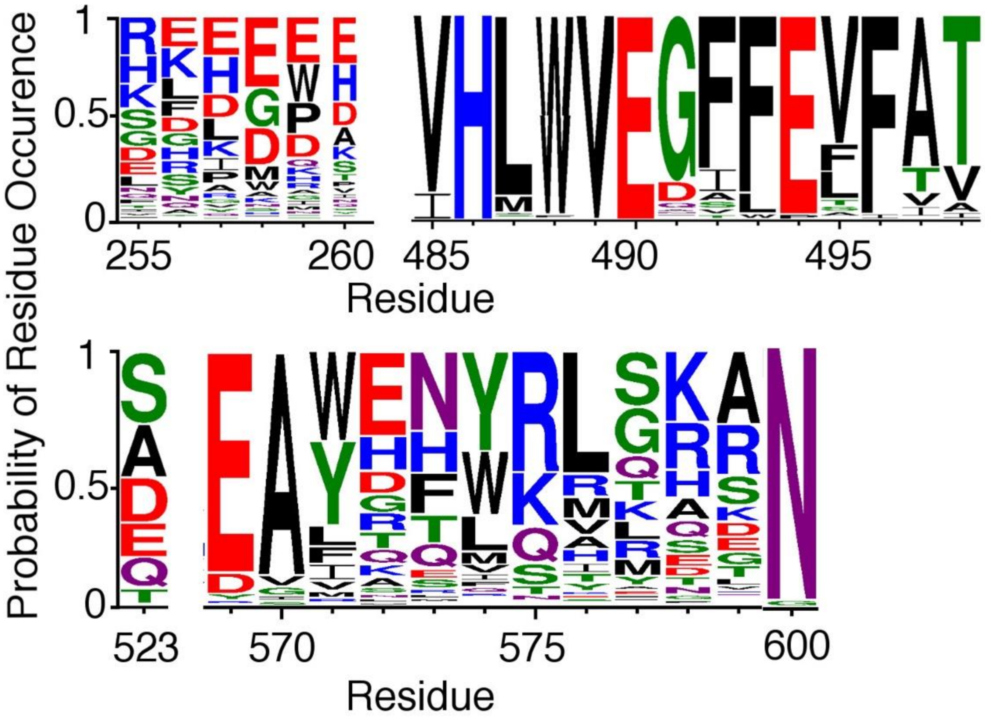

Fig. S6. Residue probability chart of qNORs. Chart indicating the probability of a certain residue appearing amongst the qNORs (224 sequences used). Selected residues are those tested by mutagenesis in this study and residues lining the putative proton transfer channel.

Table S1. Cryo-EM data collection parameters and refinement statistics.

**Data Collection & Processing**

Parameters	**Wildtype *Axq*NOR (EMDB-4618) (PDB 6QQ5)**	**Val495Ala *Axq*NOR (EMDB-4619) (PDB 6QQ6)**
Microscope and Detector	Titan Krios with K2 Summit (Gatan)	Titan Krios with K2 Summit (Gatan)
Voltage (kV)	300	300
Magnification	75,000x	47,710x
Pixel size (Å)	1.07	1.05
Defocus (µm)	-1.5 to -3.5	-1.2 to -3
Total dose (e^-/A^2)	65	49
No. of frames	40	40
Exposure time per frame	0.3	0.3
Dose per frame ($e^-/Å^2$)	1.63	1.23
No. of micrographs	3,213	1,803
Total autopicked particles	707,246	614,931
Particles in final refinement	56,134	227,359
**Refinement**		
Starting Model	*Gs*qNOR (PDB Accession Code: 3AYF)	Wildtype *Ax*qNOR (this work)
Symmetry	C2	C2
Map Sharpening B-factor ($Å^2$)	-202	-147
FSC Threshold	0.143	0.143
Map Resolution* (Å)	3.9	3.3
Model Composition		
Non-hydrogen atoms	11,829	12,123
Protein residues	1,476	1,486
Ligands	8	13
B factors ($Å^2$)†		
Protein	45.73	47.13
Ligand	40.02	40.23
Water	-	37.86
R.M.S Deviations		
Bond lengths (Å)	0.007	0.012
Bond angles (°)	1.059	1.15
Validation		
Clash score	4.89	4.40
MolProbity score	1.76	1.69
Poor rotamers (%)	0	0
Ramachandran Plot		
Favoured (%)	91.42	92.44
Allowed (%)	8.58	7.56
Outliers (%)	0	0

*Resolution determined by *RELION 3.0* †Average *B* factor values from *Phenix.real_space_refine*

Table S2. *Ax*qNOR putative proton transfer channel site-directed mutants' conservation and relative activities. Selected variants showed the most deleterious

effects on NO reduction activity of all the variants produced in this study. Samples were tested in triplicate under anaerobic conditions, using 0.2 µM final concentration of qNOR.

**Variant**	**% Conservation***	**% Activity vs WT**
E490A	100	0
E494A	99	5
N600A	99	3
S523A^	30	30

***Based on sequence alignment of 224 qNOR sequences. For E494 and N600, 99 %, conservation (less than 100%) may arise from factors such as alignment inaccuracies due to varying gaps.

^ Sequence comparison reveals that S523 is mostly replaced by an ionisable residue (Asp, Glu, Gln and Thr). There are around 25% of NORs where there is an Ala in this position which would still yield a functional NOR but their relative activity compared to AxqNOR is not unknown but could be expected to be lower than wt*Ax*qNOR.
